# Septic arthritis due to *Corynebacterium propinquum*: first report of isolation from synovial fluid in a native joint

**DOI:** 10.1128/asmcr.00138-25

**Published:** 2026-01-21

**Authors:** Rudolf Kotula, Anna Kotula, Nagarjuna Reddy Cheemarla

**Affiliations:** 1 Department of Infectious Diseases, Methodist Hospital 23511, Omaha, Nebraska, USA; 2 Department of Microbiology, Immunology & Pathology, Des Moines University 2947, West Des Moines, Iowa, USA; 3 Department of Pathology, Methodist Hospital 23511, Omaha, Nebraska, USA; Rush University Medical Center, Chicago, Illinois, USA

**Keywords:** *Corynebacterium propinquum*, septic arthritis, synovial fluid culture, native joint infection, enrichment blood culture, rare pathogen, MALDI-TOF MS, gram-positive bacilli

## Abstract

**Background:**

Septic arthritis of native joints remains an uncommon but serious clinical condition affecting approximately two per 100,000 individuals annually. The synovial membrane, being highly vascular and lacking a protective basement membrane, is particularly susceptible to hematogenous bacterial seeding, especially in patients with pre-existing joint disease. *Corynebacterium propinquum*, typically regarded as a respiratory commensal, has rarely been implicated in deep-seated infections.

**Case Summary:**

We report the first known case of *C. propinquum* isolated from synovial fluid in a patient with native knee septic arthritis. The 59-year-old female had a history of bilateral knee osteoarthritis and recent intra-articular corticosteroid injections. Initial synovial fluid cultures were negative; however, enrichment using aerobic blood culture bottles, followed by subculture, yielded *C. propinquum* identified by MALDI-TOF MS. The organism grew on aerobic blood agar but not on anaerobic media or thioglycolate broth. The patient was successfully treated with intravenous daptomycin, followed by ceftriaxone, with subsequent clinical improvement.

**Conclusion:**

This case highlights the diagnostic value of using enrichment culture and comprehensive laboratory-clinical correlation to recognize clinically significant, low-virulence organisms, such as *Corynebacterium propinquum*. Accurate species-level identification and thoughtful interpretation within the clinical context are essential to distinguish contamination from true infection, particularly in sterile-site cultures associated with native joint infections.

## INTRODUCTION

Septic arthritis is a medical emergency that can result in rapid joint destruction and permanent disability if not promptly diagnosed and treated. While *Staphylococcus aureus* and *Streptococcus* species are the most frequently isolated pathogens, infections due to *Corynebacterium* species are rare and often overlooked because they are typically considered commensal skin flora. Among these, *Corynebacterium propinquum*, a species commonly found in the upper respiratory tract, has infrequently been reported as a cause of invasive disease. We describe the first known case of *C. propinquum* septic arthritis in a native joint and review the relevant literature.

## CASE PRESENTATION

A 59-year-old woman with a history of severe bilateral knee osteoarthritis presented with acute worsening of right knee pain, swelling, and restricted range of motion. Her medical history included multiple intra-articular corticosteroid injections, reportedly over 10, with the most recent administered 2 weeks prior to the symptom onset. She had also returned from travel to Mexico within the past month. Despite chronic joint issues, she remained ambulatory and had returned to her orthopedic specialist due to progressive symptoms. Physical examination revealed significant right knee effusion, warmth, erythema, and tenderness with limited flexion. Laboratory studies showed leukocytosis (peak white blood cell 12.2 × 10^3^/μL; reference range 4–11 × 10^3^/μL), elevated C-reactive protein (92.6 mg/L; reference range 0–5 mg/L), and platelet count of 477,000/μL (reference range 150–450 × 10^3^/μL). Antinuclear antibodies were negative. Ultrasound of the lower extremity ruled out deep vein thrombosis.

Three synovial fluid specimens were available for microbiologic evaluation. Two aspirates were obtained in the outpatient orthopedic clinic several days apart, and a third specimen was collected intraoperatively at the time of arthroscopic irrigation and debridement. All three specimens underwent identical laboratory processing.

Arthrocentesis revealed elevated synovial white blood cell counts (ranging between 14,180 and 30,090 cells/μL; reference range 0–200 cells/μL), with uric acid crystals noted on one occasion. Direct Gram stains showed many white blood cells but no organisms. Each specimen was processed according to our institutional synovial fluid culture protocol. Fluid was inoculated onto Remel (Lenexa, KS) tryptic soy agar with 5% sheep blood (TSA-5%) and chocolate agar (both 35°C, 5% CO_₂_), MacConkey agar and thioglycolate broth (35°C**,** ambient air)**,** and an anaerobic CDC blood agar plate (Remel) incubated at 35°C in an anaerobic jar/bag. In addition, when volume permitted, equal aliquots (1–10 mL per bottle; minimum 1 mL) were inoculated into BD Bactec Plus Aerobic F and BD Bactec Lytic Anaerobic F bottles and monitored on the BD BACTEC System; specimens ≤3 mL were cultured only on solid media. Plates were held as follows: aerobic blood agar 3 days**,** anaerobic blood agar 5 days**,** and thioglycolate broth 5 days with subculture as indicated. Blood culture bottles were incubated per instrument protocol (up to 5 days). In all three samples, only the aerobic blood culture bottle(s) flagged positive. No growth was observed on direct plates, thioglycolate broth, or anaerobic cultures. The aerobic blood-culture bottles became positive at 2, 3, and 4 days, respectively, yielding a median time-to-positivity of 3 days (range, 2–4 days). Blind Gram stains of the anaerobic media confirmed the absence of growth.

**Fig 1:**
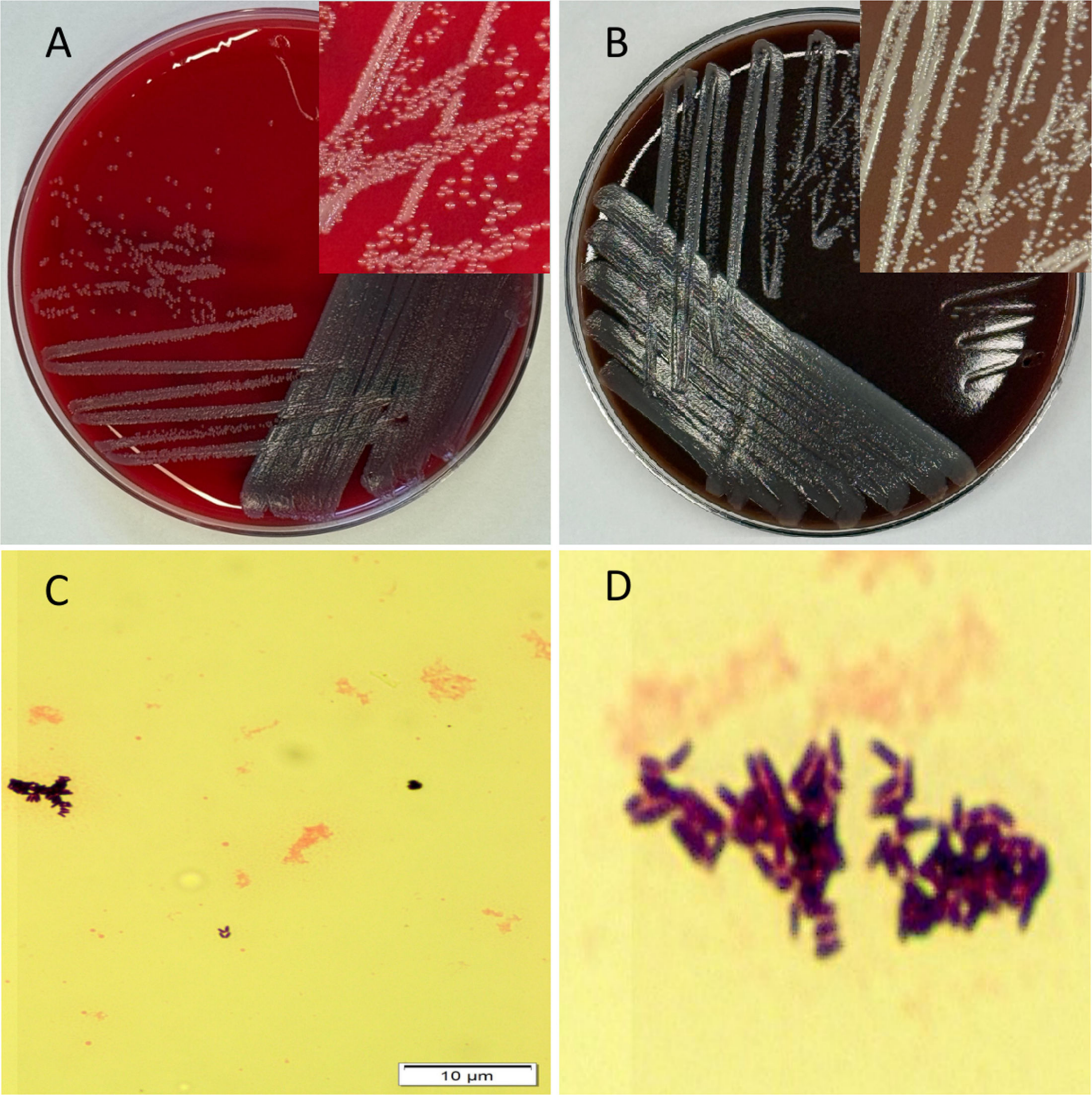
Morphology and growth characteristics of *C. propinquum*. (**A**) Growth on blood agar under aerobic conditions showing small gray, non-hemolytic colonies. (**B**) Growth on chocolate agar under aerobic conditions from the stocked isolate. Inset: magnified view of colony morphology. (**C**) Gram stain of the isolate (100× magnification). (**D**) Zoomed-in view of the Gram stain highlighting palisading coryneform bacilli arranged in a classic picket-fence/pickup-stick arrangement.

Subcultures from the positive aerobic blood culture bottles yielded small, non-hemolytic gray colonies on blood agar after 48 h of incubation in 5% CO_₂_. No anaerobic growth was observed. Identification was performed using the Bruker MALDI Biotyper CA System (Bruker Daltonics GmbH, Germany) operating with the FDA-cleared reference library claim 4 (MALDI Biotyper CA System software), which yielded a high-confidence identification of *Corynebacterium propinquum* (score 2.13). No RUO database or Vitek MS System was used, and confirmatory gene sequencing was not performed. Gram stain of the isolate showed club-shaped, palisading gram-positive bacilli in a characteristic picket-fence/pickup-sticks arrangement consistent with *Corynebacterium* spp. morphology. For documentation purposes, representative colony morphology was photographed from a fresh subculture of the archived isolate (Fig. 1).

The isolate was referred to ARUP Laboratories (Salt Lake City, Utah) for antimicrobial susceptibility testing by broth microdilution (Sensititre System from Trek Diagnostics), with results interpreted using CLSI M45 (3rd edition) breakpoints for *Corynebacterium* species. The organism was susceptible to ceftriaxone (MIC 0.12 µg/mL), doxycycline (≤0.25 µg/mL), gentamicin (≤2 µg/mL), linezolid (0.5 µg/mL), meropenem (≤0.016 µg/mL), penicillin (≤0.03 µg/mL), and vancomycin (0.5 µg/mL) and resistant to clindamycin (≥4 µg/mL), erythromycin (4 µg/mL), and trimethoprim-sulfamethoxazole (≥4/76 µg/mL). Levofloxacin MIC was 0.5 µg/mL; no categorical interpretation was available because CLSI M45 does not provide breakpoints for fluoroquinolones in *Corynebacterium* species. Detailed MIC results are summarized in Table 1.

**TABLE 1:** Antimicrobial susceptibility profile of the *Corynebacterium propinquum* isolate

Antimicrobial agent	MIC (µg/mL)	Interpretation^*a*^
Ceftriaxone	0.12	Susceptible
Clindamycin	≥4	Resistant
Doxycycline	≤0.25	Susceptible
Erythromycin	4	Resistant
Gentamicin	≤2	Susceptible
Levofloxacin	0.5	Not reported
Linezolid	0.5	Susceptible
Trimethoprim–sulfamethoxazole	≥4/76	Resistant
Meropenem	≤0.016	Susceptible
Penicillin	≤0.03	Susceptible
Vancomycin	0.5	Susceptible

^*a*^ Interpretations are based on CLSI M45, 3rd edition, breakpoints for *Corynebacterium species*. No categorical interpretation is provided for levofloxacin because CLSI M45 does not assign breakpoints for levofloxacin for *Corynebacterium* spp.

Figure 1 illustrates the growth and morphology of the isolate. Panels A and B show colony morphology (gray colonies) on aerobic blood agar and chocolate agar from a fresh subculture of the archived isolate (*inset*: magnified view); Panel C demonstrates the Gram stain morphology with typical coryneform bacilli; and Panel D shows a magnified view of palisading bacilli arranged in a characteristic picket-fence/pickup-sticks pattern.

The patient was evaluated in the Outpatient Infectious Diseases Clinic, after which she was admitted the following day for surgical management. She underwent right knee arthroscopic irrigation and debridement on the day of admission. Intravenous daptomycin (6 mg/kg every 24 h) was initiated at that time and continued for approximately 2 weeks with weekly creatine kinase monitoring, all of which remained within reference limits. Therapy was then transitioned to intravenous ceftriaxone (2 g every 24 h) to complete a total of 6 weeks of parenteral antimicrobial treatment. She experienced full symptom resolution and returned to baseline joint function by the end of therapy.

## DISCUSSION

*Corynebacterium propinquum* is a non-diphtherial, gram-positive bacillus that resides primarily in the upper respiratory tract and has historically been regarded as a commensal organism. In recent years, however, it has emerged as a potential opportunistic pathogen, particularly in immunocompromised hosts or when isolated from normally sterile body sites (1–7).

From a microbiologic standpoint, *Corynebacterium propinquum* belongs to the group of nondiphtherial corynebacteria that are typically *nonlipophilic and nonfermentative*, consistent with descriptions of the *C. pseudodiphtheriticum* group (8–10). This contrasts with clinically important *lipophilic, nonfermentative* species, such as *C. jeikeium* and *C. urealyticum*, which require lipid supplementation, grow slowly, and are frequently multidrug-resistant (8–10). In comparison, *C. striatum* is nonlipophilic but *fermentative* and has emerged as a multidrug-resistant nosocomial pathogen, including as a documented cause of bone and joint infections and native-joint septic arthritis (11). By contrast, *C. minutissimum* is nonlipophilic and fermentative but is primarily associated with superficial skin infections, such as erythrasma, rather than invasive disease (8–10). Within this broader taxonomic context, published invasive *C. propinquum* isolates have generally demonstrated broader antimicrobial susceptibility than *C. jeikeium* or *C. striatum*, which aligns with the relatively susceptible phenotype observed in our case.

In this report, we describe the isolation of *C. propinquum* from the synovial fluid of a patient with native joint septic arthritis, a highly unusual and, to our knowledge, previously unreported clinical scenario. Importantly, the organism was isolated consistently from three separate synovial fluid specimens, each yielding growth solely in aerobic blood culture bottles, while direct plating and thioglycolate enrichment remained negative. This pattern suggests a low organism burden or a degree of fastidiousness not typically attributed to *C. propinquum* and highlights the clinical utility of inoculating synovial fluid into blood culture bottles to increase the diagnostic yield.

In fact, the preferential recovery of this isolate from aerobic blood culture bottles aligns with published evidence supporting the use of blood culture systems as enrichment media for synovial fluid. Multiple studies have demonstrated that inoculating synovial fluid into blood culture bottles increases the diagnostic yield for both native and prosthetic joint infections by improving recovery of fastidious or low-inoculum organisms. Font-Vizcarra et al. showed that blood culture flasks significantly enhanced recovery of pathogens from synovial fluid in prosthetic joint infections compared with direct plating alone (12). Similarly, the 2018 International Consensus Meeting (ICM) on Musculoskeletal Infection recommends inoculation of synovial fluid into aerobic and anaerobic blood culture bottles to improve sensitivity, especially when only small volumes are available (13). These data support our findings and reinforce that enrichment systems can be critical for detecting uncommon pathogens, such as *Corynebacterium propinquum* (12–14).

The repeated isolation of the same organism from a sterile site supports its etiologic significance in this case. While coryneform bacteria are often considered contaminants, especially when isolated from non-sterile sites, the context here is critical. The organism was recovered from a sterile compartment on multiple occasions in a symptomatic patient with classic clinical findings of septic arthritis. Such concordant evidence strengthens the case for true infection rather than colonization or contamination (1–7).

Our isolate of *C. propinquum* demonstrated a generally favorable susceptibility profile (ceftriaxone 0.12 µg/mL, penicillin ≤0.03 µg/mL, vancomycin 0.5 µg/mL) consistent with previously published cases of this species in invasive infections, which also reported broad susceptibility to β-lactams and glycopeptides (1, 3, 5, 7, 15). In contrast, *C. striatum* and other nondiphtherial *Corynebacterium* species have emerged as frequently multidrug-resistant pathogens, with decreased susceptibility to many β-lactams, macrolides, fluoroquinolones, and trimethoprim-sulfamethoxazole, although vancomycin largely retains activity (15, 16). Hence, while our patient’s isolate demonstrated an ‘expected’ phenotype for *C. propinquum*, this underscores the importance of species-level identification and AST given the broader genus may not behave the same way. Given the increasing recognition of resistance among nondiphtherial corynebacteria, susceptibility testing is warranted when such organisms are recovered from sterile sites.

From a diagnostic standpoint, Gram stain morphology was also informative: palisading, club-shaped gram-positive bacilli arranged in irregular, angular clusters provided a strong presumptive clue, reinforcing the importance of early microscopy in guiding organism workup. Growth on blood agar, along with lack of anaerobic growth, was consistent with the organism’s known aerobic physiology.

The recovery of this organism was made possible by modern species-level identification techniques. While tools, such as MALDI-TOF MS, are now routine in clinical microbiology laboratories, their broad implementation has greatly improved recognition of previously underappreciated or misidentified species as potential pathogens. *C. propinquum* may have been similarly overlooked in past cases of septic arthritis before the adoption of these technologies.

To place our case in context, we reviewed prior reports of *C. propinquum* infections (Table 2). Although uncommon, these infections have occurred across diverse clinical settings and syndromes, suggesting that the organism’s rarity likely reflects underrecognition rather than true scarcity. Reported cases collectively support its ability to cause disease in both immunocompromised and immunocompetent hosts when isolated from sterile body sites.

**TABLE 2:** Summary of reported *Corynebacterium propinquum* human infections

Case	Reference	Country	No. of patients	Age (yrs)	Sex	Clinical syndrome	Risk/host factors	Culture source	Identification method	Treatment	Outcome
1	Jangda et al. (1)	USA	1	48	M	Prosthetic mitral-valve endocarditis	Prosthetic valve; RA on prednisone	Blood culture	Phenotypic methods (MALDI-TOF not reported)	Empiric vancomycin, cefepime, gentamicin → targeted ceftriaxone	Survived
2	Kuriakose et al. (3)	USA	1	63	M	Native-valve endocarditis	T2DM, COPD, brain infarction	Blood culture	NR	Empiric vancomycin → ceftriaxone (per susceptibility)	Survived
3	Motomura et al. (2)	Japan	3	NT^*a*^	NT^*a*^	Respiratory infections	Elderly adults (per abstract)	Sputum	Biochemical testing (pre-MALDI era)	NT^*a*^	NT^*a*^
4	Kawasaki et al. (5)	Japan	1	7	F	Native tricuspid-valve infective endocarditis	Ventricular septal defect (VSD), ventriculoatrial communication, and minor dental procedure 20 days before onset (no prophylaxis)	Blood culture	API Coryne biochemical panel, 16S rRNA gene sequencing, partial *rpoB* gene sequencing, MALDI-TOF MS	Ceftriaxone + gentamicin → ampicillin + gentamicin; cefazolin + gentamicin for IE	Clinical cure after prolonged therapy
5	Fernández-Vecilla et al. (4)	Spain	1	64	M	Late-onset endophthalmitis	T2DM, proliferative diabetic retinopathy w/ diabetic macular edema	Aqueous humor, vitreous humor, and vitrectomy cassette	16S rDNA sequencing	Intravitreal vancomycin + ceftazidime and intracameral cefuroxime at presentation; systemic oral ciprofloxacin plus topical moxifloxacin and dexamethasone/tobramycin eye drops	Partial recovery of vision
6	Megdich et al. (7)	Tunisia	1	52	M	Native-valve infective endocarditis	Acute articular rheumatism	Blood cultures	Biochemical tests + 16S rRNA sequencing	Vancomycin → valve surgery	Survived
7	Yukari et al. (6)	Japan	1	84	F	Microbial keratitis after AM transplantation	AM graft; chronic ocular disease; steroid drops	Corneal scraping	MALDI-TOF MS (score 2.371)	Gentamicin + gatifloxacin eye drops	Improved (vision unchanged)
8	Present case (this report)	USA	1	59	F	Native knee septic arthritis	Repeated intra-articular steroid injections	Synovial fluid (aerobic bottle only)	MALDI-TOF MS (FDA-cleared library)	Daptomycin → ceftriaxone	Full recovery

^*a*^ NT, not translatable from original Japanese-language article. Only information provided in the English abstract or figure legends was used. NR, not reported.

This case underscores the diagnostic challenges posed by unusual or fastidious organisms recovered from sterile fluids such as synovial aspirates. *C. propinquum*, while often regarded as a commensal, can cause clinically meaningful infections when isolated repeatedly in an appropriate clinical context. The use of enrichment culture and MALDI-TOF MS facilitated accurate detection and timely identification. Greater awareness, continued case reporting, and standardized susceptibility testing will be essential to define its epidemiology, virulence potential, and antimicrobial resistance patterns.

In conclusion, our findings contribute to the growing evidence that non-diphtherial *Corynebacterium* species, including *C. propinquum*, should not automatically be dismissed as contaminants. Rather, their presence in multiple concordant cultures from a compatible clinical syndrome should prompt careful evaluation and correlation with host factors and infection site.
